# In Vivo Transfection of Rat Salivary Glands With Fluorescently Tagged Aquaporin-5 Channel DNA

**DOI:** 10.7759/cureus.24555

**Published:** 2022-04-28

**Authors:** Sanjib Adhikary, Max Hennessy, David Goldrich, Victor Ruiz-Velasco, Timothy K Cooper, Neerav Goyal

**Affiliations:** 1 Anesthesiology and Perioperative Medicine, Penn State Health Milton S. Hershey Medical Center, Hershey, USA; 2 Otolaryngology - Head and Neck Surgery, Penn State Health Milton S. Hershey Medical Center, Hershey, USA; 3 Pathology, Penn State Health Milton S. Hershey Medical Center, Hershey, USA

**Keywords:** saliva, gene delivery, in vivo transfection, aquaporin channels, salivary glands

## Abstract

Background

The acinar cells of salivary glands are responsible for most saliva production and are, unfortunately, h­­ighly radiosensitive. As such, dry mouth or xerostomia is an adverse effect experienced by half of head and neck cancer patients treated with radiation. We evaluate a novel method of gene transfection of aquaporin channels to rat salivary glands.

Materials and methods

A green fluorescent protein (GFP)-tagged human Aquaporin-5* (AQP5)* cDNA sequence cloned into a pCMV6-AC-GFP vector was complexed with lipofectamine 2000. One submandibular gland of the anesthetized rats was injected with the complexed cDNA and lipid solution under ultrasound guidance, while the opposite gland was injected with the vehicle control. The animals were sacrificed between 24 to 48 hours post-injection. The salivary glands were removed and evaluated via fluorescence imaging. Western blot assays were also performed to determine *AQP5* cDNA expression.

Results

In the experiments, the submandibular glands were identified and injected under ultrasound guidance. Four control glands and eight experimental glands were evaluated. The cDNA was expressed successfully and variably within the experimental glands, noting greater intensity along the cell surface consistent with appropriate trafficking of the *AQP5* channel. Western blot analysis demonstrated variable expression in the experimental sample with no expression in the control sample. Several glands across the groups showed mild to moderate interstitial edema or inflammation.

Conclusion

In this study, we demonstrate an alternative in vivo transfection method via lipofection and demonstrate the successful expression of the AQP5 channel in rat salivary gland tissue.

## Introduction

Saliva plays a number of critical roles in dental health, including mouth lubrication, infection prevention, food bolus preparation, and lubrication during swallowing [1]. Salivary gland tissue contains acinar, ductal, and myoepithelial cells, with the secretory tissue consisting of 80% acinar cells and 20% ductal cells. The acinar cells are responsible for the majority of exocrine protein secretion and the only site of water movement [2]. Further, the acinar cells are highly radiosensitive, and salivary flow diminishes to approximately 20% of pre-treatment levels following radiation therapy [3]. As such, xerostomia, or dry mouth, is a common adverse effect experienced by the approximately 600,000 new head and neck cancer patients annually worldwide [4]. Treatment options for radiation-induced xerostomia are extremely limited and include cytoprotectants, saliva stimulants, oral hydrating products, salivary gland transfer, acupuncture, and hyperbaric O_2_ therapy [5]. Unfortunately, these treatments only offer transient symptom relief and no definitive long-term treatment is available, making this adverse effect of treatment debilitating to a cancer survivor’s quality of life. Gene therapy represents a novel treatment option that may restore the glands’ ability to secrete saliva through the heterologous expression of aquaporin channels in the salivary gland tissue.

Aquaporins (AQPs) are transmembrane protein channels capable of moving water across the cell membrane in the presence of an osmotic gradient. In rats, there are six known aquaporins expressed in mammalian salivary gland tissue, AQP1, AQP3-AQP6, and AQP8 [6]. Of these proteins, AQP5 is the only channel expressed on the apical membrane of acinar cells in both humans and rats [7]. Due to its location on the apical membrane of the secretory acinar cells, AQP5 plays a major role in water movement saliva secretion. This was demonstrated in aquaporin-5 knockout mice, which produced 60% less saliva that was also more viscous and hypertonic when compared to normal mice [8-9].

Most current experimental gene transfer techniques utilize viral vectors to deliver aquaporin DNA constructs to salivary gland tissue. Multiple studies have employed strains of recombinant adenovirus vectors to deliver the human *AQP1 *gene to irradiated rat and pig salivary glands [10-12]. While these treatments increased saliva flow, the virally induced transgene expression was short-lived and salivary flow subsequently returned to the pre-treatment baseline. Unfortunately, subsequent viral-mediated transfection is attenuated by the human immune response to the viral capsid, and aquaporin expression is greatly reduced [10-13]. Given the promising initial results of these studies, a compelling need for non-viral gene transfer techniques exists. The most effective of the non-viral vectors reported in the literature, to our knowledge, is the ultrasound-assisted non-viral gene transfer (UAGT) method, which utilizes lipid/perflutren microbubbles coated with aquaporin cDNA to deliver the payload to mouse and pig salivary gland tissue [14-15]. This method does not elicit an extracellular host immune response (cellular or humoral immunity) and as such subsequent vector administrations are not attenuated. In a study by Wang et al., both UAGT and adenoviral-mediated gene transfer were performed in parallel, and the efficacy in delivering the aquaporin cDNA to salivary gland tissue was found to be similar [15].

While previous studies expressed the AQP1 channel subtype, AQP5 is the only subtype that appears to play a major role in saliva secretion and is the only AQP shown to be expressed in both acinar and ductal cells [5]. As such, we elected to use *AQP5 *cDNA construct as a candidate aquaporin to transfect rat salivary glands in vivo. In this study, we describe a technique with green fluorescent protein (GFP)-tagged *AQP5* cDNA complexed with lipofectamine to successfully express the AQP5 channel in rat submandibular salivary gland tissue within 24 hours. 

This article was previously presented as a poster session at the 2019 American Head & Neck Society (AHNS) Annual Combined Otolaryngology Spring Meetings (COSM) meeting on May 1, 2019, in Austin, Texas.

## Materials and methods

Animals and cDNA injection

The experiments performed were approved by the Penn State College of Medicine Institutional Animal Care and Use Committee (41949) and complied with the National Institutes of Health guidelines. Male Sprague-Dawley rats (Crl:SD, strain code 400, Charles River Laboratories, Wilmington, Massachusetts, United States) were used following a minimum acclimation period of seven days in a light-controlled room (12-hour light/12-hour dark cycle) with ad libitum access to standard rat chow and water. In this small pilot study, 14 male rats were employed. Female rats were not included due to the variable nature of female data caused by hormonal fluctuations associated with the female reproductive cycle.

cDNA construct and lipid complexing

The GFP-tagged human *AQP5* cDNA construct was cloned into the pCMV6-AC-GFP vector (Origene Technologies, Inc., Rockville, Maryland, United States) and stored in Tris-Ethylenediamine tetraacetic acid (TE) buffer. On the day of the transfection, the cDNA construct (25 µg) was complexed with lipofectamine 2000 (25 µl) in 100 µl Opti-MEM® (Thermo-Fisher Scientific, Waltham, Massachusetts, United States) for approximately 15 minutes. Afterward, the solution (final volume 150 µl) was drawn up to a 1cc syringe with a 30-gauge needle. Thereafter, the rats were anesthetized with 3% isoflurane and ultrasound imaging was employed to visualize the submandibular salivary glands. The ultrasound machine (Fujifilm Sonosite M-Turbo, FUJIFILM Sonosite, Inc., Bothell, Washington, United States) employed was equipped with a 6-13 MHz transducer. Once the glands were located, the cDNA lipid mixture (25 µg cDNA/gland) was injected into the gland. The contralateral side (control) was injected with lipofectamine 2000 alone (final volume 150 µl). The animals were sacrificed at various time points between 24 to 48 hours post-injection. The salivary glands were then removed and frozen in optimal cutting temperature media (OCM) for fluorescence imaging or fixed in 10% neutral buffered formalin followed by paraffin processing and embedding and stained with hematoxylin and eosin (H&E) staining for histopathological evaluation.

Fluorescence imaging

The salivary gland fluorescence images were obtained with a Nikon TE2000-U microscope (Nikon Corporation, Minato City, Tokyo, Japan) employing 10X, 20X, and 40X objectives, an Orca-ER 1394 CCD camera (Hamamatsu Photonics K.K., Hamamatsu City, Japan), PhotoFluor® ii (89 North, Williston, Vermont, United States) for illumination, and iVision software (Biovision Technologies, Exton, Pennsylvania, United States). The images were obtained with a filter set (B-2E/C, Nikon) containing an excitation filter at 480+15 nm, a dichroic beam splitter of 505 nm (LP), and an emission filter at 535+20 nm. The images shown were pseudo-colored.

Histopathological assays

In this set of experiments, histopathological analysis was performed on H&E-stained salivary glands injected with either lipid alone or lipid complexed with *AQP5* cDNA. The glands were collected 24 hours post-injection and fixed in 10% neutral buffer formalin. Thereafter, the glands were sectioned and stained. The American College of Veterinary Pathologists diplomate performing the analysis was blinded to the identification of the experimental groups.

Western blotting analysis

Western blotting assays of salivary gland tissue were performed to determine GFP-tagged AQP5 protein levels employing the Wes system (Protein Simple, San Jose, California, United States) as previously described [16]. Briefly, salivary gland protein was isolated employing the NucleoSpin Protein Kit (Macherey-Nagel, Bethlehem, Pennsylvania, United Sates). Thereafter, the protein samples were prepared for Western blotting. The microplates were loaded with 1.4 µg/µl protein per lane. The secondary antibody and reagents were provided by the manufacturer (Protein Simple). The anti-GFP antibody (Abcam, Waltham, Massachusetts, United States; Cat. No. ab290) was employed at 1:1000 dilution.

## Results

**Figure 1:**
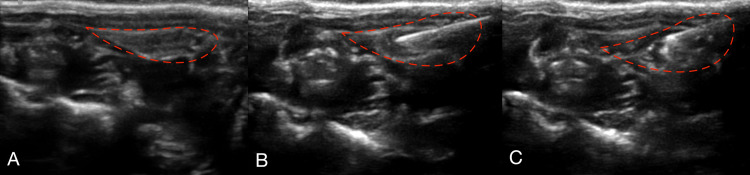
Ultrasound-guided delivery of Aquaporin-5 DNA in rat submandibular gland: (A) Ultrasound image of submandibular gland (outlined in red) prior to needle insertion; (B) Image showing insertion of a 30-gauge needle into the bulk of the gland; (C) The solution containing the cDNA lipid complex was injected and visualized, indicated by the bright echo area.

**Figure 2:**
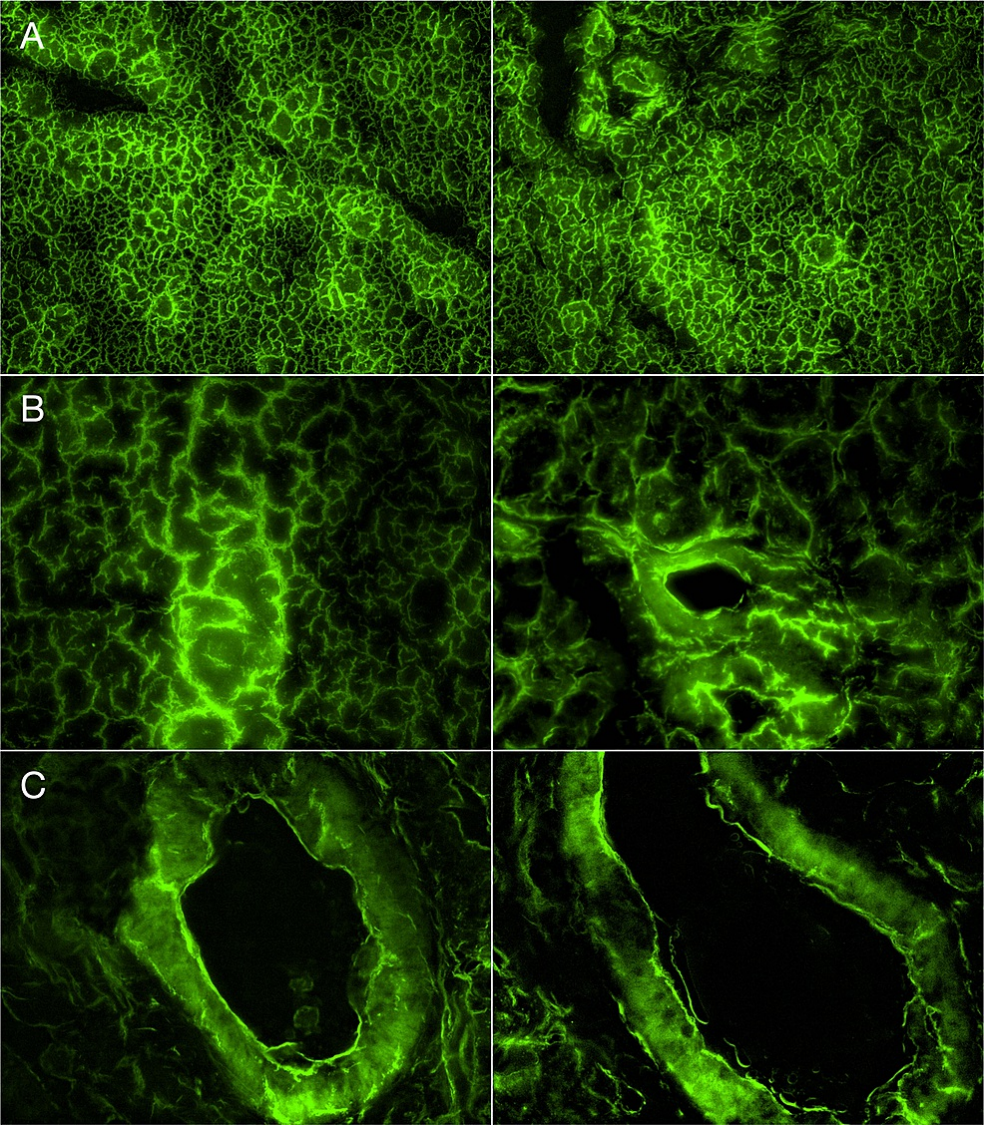
Detection of GFP-tagged AQP5 channel expression in rat salivary glands as revealed by fluorescence imaging Immunofluorescence images of a rat salivary gland transfected with 25 µg of GFP-tagged *AQP5* cDNA 24 hours post-injection. The left and right columns represent two different regions of the salivary gland. The images shown were acquired using 10X (2A) 20X (2B) and 40X (2C) objectives with a filter set containing an excitation filter at 480 nm and emission filter at 535 nm. The images were pseudo-colored. GFP: Green Fluorescent Protein; AQP5: Aquaporin-5

In the present study, we sought to examine whether rat salivary glands could be transfected in vivo by employing lipofection. Ultrasound imaging was employed to locate the submandibular glands (Figure 1A). Figure 1 shows images before (Figure 1A) and during (Figure 1B) the injection of the lipid: cDNA mixture into the submandibular gland using a 30-gauge needle. Figure 2 depicts the successful expression of GFP-tagged AQP5 channels in the rat salivary gland 24 hours post-injection. Figure 2A shows that expression levels varied within the tissue. This was expected given that some areas likely received more of the lipid: cDNA solution. Additionally, Figure 2 (B, C) also indicates that the fluorescence intensity is greater on the cell surface compared to the cytosolic fluorescence intensity, indicative that the AQP5 channels are appropriately trafficked to the cell membrane.

**Figure 3:**
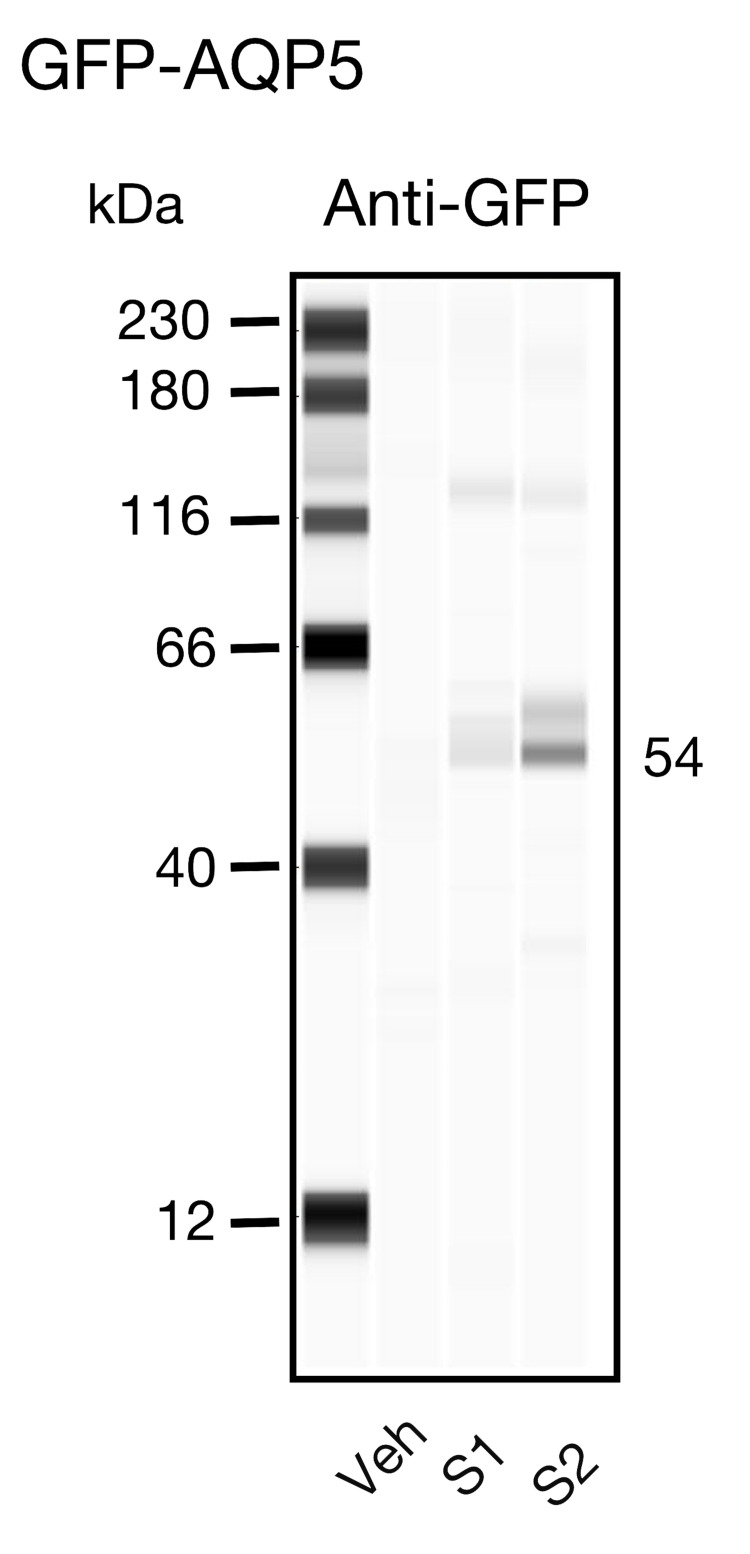
Western blotting detection of GFP-tagged AQP5 in rat salivary glands Western blots for GFP in rat salivary gland tissue sections isolated 48 hours post transfection with either vehicle (Veh) or GFP-tagged *AQP5* cDNA (S1 and S2). S1 and S2 represent samples randomly picked from the entire tissue. Each lane was loaded with 1.4 µg/µl. The anti-GFP antibody was used at a dilution of 1:100. GFP: Green Fluorescent Protein; AQP5: Aquaporin-5

In a separate set of experiments, we isolated vehicle- and cDNA-transfected salivary gland tissue 48 hours post-injection to determine the expression of GFP-tagged AQP5 channels. Figure 3 shows a Western blot for GFP in a control (Veh) and two cDNA transfected samples (S1 and S2) randomly obtained from the same gland. The bands (~ 54 kDa) represent the approximate molecular weight for GFP-tagged AQP5. Like the results described in Figure 2, it can be observed that in both samples from the GFP-tagged *AQP5* transfected gland the expression levels varied. On the other hand, GFP was not detected in the control group.

**Figure 4:**
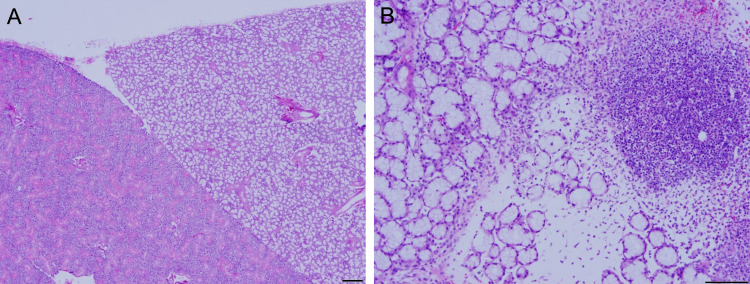
Histological analysis of hematoxylin and eosin-stained rat salivary glands Representative images of vehicle-injected (4A) and cDNA‑injected (4B) rat salivary gland tissue. The image shown in 4A shows normal submandibular (left) and sublingual (right) tissue, while inflammation and edema are present in the cDNA-injected tissue. Scale bar is 200 µm.

The final set of experiments was undertaken to examine morphologic changes following injection of the salivary glands. In the vehicle-injected group, three of the four glands exhibited normal characteristics while one displayed moderate edema and inflammation (Figure 4). Contrastingly, in the *AQP5* cDNA-transfected group, three of the eight injected glands demonstrated normal characteristics. However, mild to moderate interstitial edema or inflammation was observed in the remaining five glands. These results suggest that the needle injection may exert, in some cases, untoward effects on the gland tissue, such as inflammatory reaction.

## Discussion

The salivary gland is an ideal target for gene therapy due to its structural characteristics and the technical ease of injection. The gland is encapsulated and accessible due to its superficial anatomic location [5,13]. To date, viral vectors have provided the most efficacious method for gene therapy for xerostomia. Multiple studies have used strains of recombinant adenovirus vector to successfully transfer the human* AQP1* gene to irradiated rat and pig salivary glands [2,10-12]. When successfully expressed by the ductal cells, aquaporins increased salivary flow to 35-84% of pre-radiation levels. However, the expression is transient, with salivation levels decreasing to baseline two to four weeks after the initial injection. Furthermore, the resultant immune response following administration attenuates the efficacy of subsequent injections [2,10-12]. This is not unexpected, as unlike gene-editing techniques such as CRISPR-Cas9 (clustered regularly interspaced short palindromic repeats-CRISPR associated protein 9), viral-mediated transfection results in the transient presence of transgene DNA that exists as an episome in the host cell nucleus. Following transgene degradation, additional gene therapy administration is required for continued protein expression. Despite their success in initial administrations, the efficacy of subsequent viral vector administrations can be significantly attenuated by the immune response to the viral capsid protein [17]. This immune‑mediated attenuation in efficacy represents one of the most significant barriers to successful gene therapy in humans [10-12,17]. Furthermore, the use of viruses as a means of transduction presents safety concerns that are not insignificant, and widespread acceptance of viral-mediated gene transfer has been limited by high-profile patient deaths in testing during earlier decades [18]. Despite improved safety profiles of viral-mediated gene transfection in recent years, viral-mediated transfection carries the possibility of off-target effects, unexpected toxic effects, as well as the possibility of viral mutation or recombination, cytotoxic gene products, viral transmissibility, and potential risk to immunosuppressed patients [19].

To overcome the inherent challenges associated with viral-mediated gene therapy, non-viral vectors represent a means to achieve gene expression at therapeutic levels. Due to its high biocompatibility and transfection efficiency combined with low immunogenicity and lack of similar potential consequences, lipid-mediated gene transfection represents a viable alternative [20-21]. Lipofectamine is a proprietary formulation that contains lipid subunits, which when combined with DNA in an aqueous environment, entraps the DNA in a liposome. This allows negatively charged nucleic acids to pass freely through the cell membrane and into the cytoplasm via endocytosis [22]. Previous studies have demonstrated nephrotoxicity and hepatoxicity with systemic administration of lipofectamine [23]. Upon microscopic evaluation, GFP-tagged AQP5 channels were expressed along the cell membrane as indicated by a greater fluorescence intensity than that observed in the cytoplasm (Figure 2). Western blot analysis subsequently confirmed GFP expression in the salivary gland tissue as well (Figure 3). The efficacy of AQP5 expression in restoring salivary gland function was not measured in this study but has previously been shown to increase saliva production [2,10-11]. In regard to local tissue safety following injection, inflammation was present in both the experimental and control groups (Figure 4), suggesting that the pathological features resulted from the injection process itself rather than the lipid vehicle or cDNA construct.

While promising, this study carries some limitations. Despite the successful demonstration of AQP5 in vivoexpression within salivary gland tissue, the duration of this effect was not measured due to the sacrifice of transfected mice for immunohistopathologic examination. Similarly, while gene expression was confirmed via GFP visualization on fluorescent microscopy, transfected rats were not monitored for clinical improvement in salivation after AQP5 expression. Nonetheless, prior studies inducing AQP5 expression have shown it to increase salivary production [2,10-11]. Future work will aim to continue to develop and adapt non-viral transfection methods approved for human use that can successfully deliver *AQP5* cDNA to salivary gland tissue to potentially restore salivation following radiation therapy.

## Conclusions

Our study indicates that the AQP5 channel can be successfully expressed in rat salivary glands in vivoemploying lipid-mediated transfection under ultrasound guidance. Lipophilic methods of DNA transfection such as lipofectamine appear locally safe in salivary gland tissue, and no deleterious systemic effects were noted. Further in vitrostudy in human tissue is warranted to develop and adapt non-viral transfection methods approved for human use that can successfully deliver *AQP5* cDNA to salivary gland tissue. 
